# Barriers and Facilitators to Optimal Antibiotic Prescribing on Discharge from the Hospital to Nursing Homes

**DOI:** 10.1017/ash.2024.161

**Published:** 2024-09-16

**Authors:** Jon Furuno, Michelle Zhou, Christopher Crnich, Dominic Chan, Caitlin McCracken, Sally Jolles, Brie Noble, Jessina McGregor, YoungYoon Ham, Emily Shephard, Seiko Izumi

**Affiliations:** Oregon State University College of Pharmacy; Legacy Health; University of Wisconsin; Oregon State University; University of Wisconsin Madison Department of Medicine; Department of Quantitative Health Sciences, Mayo Clinic, Scottsdale, AZ, USA; Oregon Health & Sciences University

## Abstract

**Background:** Up to half of antibiotics used in nursing homes (NHs) are initiated in acute care hospitals prior to nursing home admission. Optimizing antibiotic prescribing on hospital discharge presents an opportunity to improve NH antibiotic use. We aimed to identify barriers and facilitators to optimal antibiotic prescribing on discharge from the hospital to NHs. **Methods:** This was a qualitative thread of a convergent parallel mixed methods study to identify high-value targets to optimize antibiotic prescribing on discharge from the hospital to NHs. We conducted 32 qualitative interviews: 16 with prescribers (9), pharmacists (6), and a care manager (1) from 3 acute care hospitals and 16 with advanced practice providers (12) and registered nurses/nurse managers (4) from 7 NHs in Oregon and Wisconsin. Interview participants were asked to describe their typical practice for prescribing antibiotics on discharge to NHs or admitting patients with antibiotic prescriptions from hospitals, and about barriers and facilitators for optimal antibiotic prescribing during these transitions. Interviews were audio recorded, transcribed verbatim, and analyzed by 3 investigators using the qualitative descriptive analysis **Results:** Hospital healthcare workers described that there are different practice flows for oral and intravenous (IV) antibiotic prescribing. IV antibiotic orders are typically routed to the infectious diseases (ID) specialists and ID pharmacists to review and verify appropriateness. There were minimal established workflows to review and verify oral antibiotic orders. Pharmacists appeared integral to optimal antibiotic prescribin; however, the high frequency of oral prescriptions and short turnaround times from discharge orders to transportation limited pharmacists’ abilities to review these orders. With limited pharmacist involvement, the quality of oral antibiotic prescription relied on the prescribers’ knowledge, and there was no systematic oversight for inexperienced prescribers or specialists who may not be familiar with the adequacy of antibiotic use for NH residents. NH participants perceived that most antibiotics prescribed from hospitals were appropriate. Yet, some commented that they occasionally observe inadequate or unusual prescriptions from newer prescribers or specialists. NH participants most common concern related to antibiotic prescriptions was missing information including unclear or lack of antibiotic indications or stop dates. NH participants also stated that the frequent need to contact the hospital to obtain missing information is challenging and burdensome. **Conclusions:** Qualitative interviews identified several barriers and facilitators to optimal antibiotic prescribing on discharge to NHs. These results will be used to develop an intervention to improve antibiotic prescribing during these transitions.

